# Unexpected Discrepancies in the Histopathological and Microbiological Diagnoses of Suspected Mucormycosis Cases at a South Indian Tertiary Care Center During the COVID-19 Pandemic

**DOI:** 10.7759/cureus.75478

**Published:** 2024-12-10

**Authors:** Rallapalli Rajyalakshmi, Sai Anirudh Athaluri, Durga R Arava, Kusa R Pyla, Sandeep Varma Manthena

**Affiliations:** 1 Pathology, Rangaraya Medical College, Kakinada, IND; 2 Medicine, Rangaraya Medical College, Kakinada, IND; 3 Microbiology, Rangaraya Medical College, Kakinada, IND

**Keywords:** aspergillus, covid 19, covid-associated mucor mycosis, culture, fungal sinusitis, histopathology, microbiology, mucor, rhino-orbito-cerebral mucormycosis

## Abstract

Introduction

Mucormycosis is an uncommon fungal infection caused by filamentous fungi of the Mucorales order, namely Rhizopus, Lichthemia, andMucor species. The incidence and prevalence of mucormycosis reached an all-time high during the COVID-19 pandemic due to excessive steroid use and other factors, leading to the coining of the term CAM (COVID Associated Mucormycosis). The diagnosis of mucormycosis is by a combination of histopathology and microbiological techniques, such as KOH mount and culture. Although microbiological and histopathological findings usually correlate, certain discrepancies are known to occur and are discussed in our study. The aim of our research is to study the correlation between histopathology and microbiology findings, as well as their respective merits and limitations, in suspected mucormycosis cases in COVID-19 patients at a tertiary healthcare center in South India.

Methods

It is a retrospective study, where data from 74 COVID-19 patients admitted in the ENT ward between June 2021 and August 2021 with a suspicion of mucormycosis was collected. Samples from these patients were sent to the pathology and microbiology departments of Rangaraya Medical College. KOH mount, culture on Saboraud's Dextrose Agar (SDA), and histopathology findings were analyzed.

Results

Histopathology and culture findings of Mucormycosis were correlated in 64 isolates (86.5%), including five cases (6.7%) of mixed infections, where the culture was positive for only a single type of fungus. In the remaining 10 isolates, discrepancies were observed, accounting for 13.5% of the total sample size. Pearson’s correlation coefficient test did not reveal any statistically significant correlation in the case of histopathology and microbiology culture or KOH mount, further highlighting the presence of discrepancies. The high correlation between KOH mount and culture isolates (100%) in our study is attributable to the processing of the same sample by the microbiology department. On the other hand, the correlation between histopathology and culture in our study is 86.5%, and the expected correlation as suggested by previous studies is only 50%. These discrepancies between culture and histopathology could be due to different samples being sent to the two departments.

Conclusion

Although fungal cultures are considered the gold standard, they have drawbacks such as slow growth, and the potential for both false positives and false negatives. KOH mount is a rapid and cost-effective method, but it lacks specificity and sensitivity. Histopathology offers the advantage of specific species identification and assessment of tissue inflammation; however, it is an invasive procedure and poses challenges in accurately identifying the fungus. A multidisciplinary approach, with coordination between physicians, surgeons, pathologists, clinical microbiologists, and radiologists, is essential for timely diagnosis and management. For accurate identification of the causative organism, it is recommended to send the same sample for histopathological examination, KOH mount, and culture.

## Introduction

Mucormycosis, also known as zygomycosis, is an uncommon fungal infection with high morbidity and mortality. It is caused by filamentous fungi of the Mucorales order, mainly Rhizopus species, Lichthemia species, and Mucor species [1]. Mucormycosis has conventionally been categorized based on the site involved into rhino-cerebral, pulmonary, cutaneous, gastrointestinal, and disseminated types, with paranasal sinus infection being the most common presentation. Its incidence has surged during the period of COVID-19 in India, predominantly owing to COVID-induced immune dysfunction and excessive use of corticosteroids resulting in immunosuppression [2]. Mucormycosis is initially suspected based on clinical features, with radiological investigations playing an important role. However, mucormycosis is difficult to diagnose by clinical examination alone as it mimics many other diseases including rhinosporidiosis, granulomatous infections, and malignancies. The final confirmatory diagnosis along with the identification of causative fungus involves a combination of histopathology, KOH mount, and microbiological culture [3]. Though microbiology and histopathology are complementary methods with their advantages and disadvantages for evaluating fungal infections, discrepancies between the two tend to occur. This novel study sheds light on such discrepancies between histopathology and microbiology findings in suspected mucormycosis cases during the COVID-19 pandemic at a South Indian tertiary healthcare center, discusses the merits and demerits of various techniques employed in diagnosis, and addresses the underlying reasons for these differences in detail. 

## Materials and methods

This study was a retrospective observational study conducted at Rangaraya Medical College, Kakinada during the COVID-19 pandemic. It was designed to evaluate the diagnostic concordance between histopathology and microbiological methods in the diagnosis and species identification of suspected rhino-orbito-cerebral and paranasal-sinus mucormycosis in COVID-19 patients. A total of 74 patients admitted to the ENT ward during the second wave of the COVID-19 pandemic were included in the data collection period, which spanned from June 2021 to August 2021.

The inclusion criteria are as follows: histopathologically confirmed cases of mucormycosis in the rhino-orbito-cerebral and paranasal-sinus regions of COVID-19 patients (diagnosed based on a COVID-19 rapid antigen test as well as RT-PCR), who were admitted to the ENT ward between June 2021 and August 2021. The patients must have had specimens sent for both histopathological and microbiological studies (including KOH mount, culture on Saboraud's Dextrose Agar (SDA), and LPCB mount) at the study institute. Additionally, only patients who provided informed consent at the time of admission for the use of their clinically relevant data (including clinical history, history of risk factors, examination findings, demographic details, treatment history, and lab reports) for research purposes were included, provided their personal identity details remained confidential. Only patients aged 18 years or older were included.

The exclusion criteria include mucormycosis infections in COVID-19-negative individuals, fungal infections other than mucormycosis, mucormycosis or other fungal infections involving organ systems other than the rhino-orbito-cerebral and paranasal-sinus regions, patients who had received prior antifungal therapy before sampling, specimens from patients with suspected mucormycosis but sent for either histopathology or microbiological studies alone, patients under 18 years of age, and those who did not provide informed consent. 

Sampling methods

Tissue samples from suspected lesions were collected through endoscopic sinus surgery, debridement, or biopsy procedures. They were fixed in 10% neutral buffered formalin, processed, and stained with hematoxylin and eosin (H&E) for histopathology examination. Periodic Acid-Schiff and Gomori's methenamine silver (GMS) stains were also used for fungal element visualization. Sterile swabs (in most cases) and occasionally, tissue samples were sent for culture in sterile containers to the microbiology laboratory wherein a KOH mount was performed on each sample, and the fungal elements were also cultured on SDA within half an hour of receiving each sample (as per institutional protocol to prevent drying of sample and loss of viability). Subsequently, lactophenol cotton blue (LPCB) staining was done to identify the isolates based on morphology.

Diagnostic protocols 

The histopathological examination focused on identifying the broad, aseptate, hyaline hyphae characteristic of Mucorales, with particular attention to angioinvasion, necrosis, and mixed fungal morphology. Cultures for microbiological studies were incubated for two weeks to promote fungal growth, and organism identification was performed based on colony morphology and microscopic examination of LPCB mounts. Initially, two pathologists (an Associate Professor and a Professor in the institute of study conduction) examined each histopathology slide before giving positive results. In instances of discordance between histopathology and culture, the respective slides were reviewed by two more pathologists (both Professors of Pathology in the same institute).

Statistical analysis

Findings from histopathology and microbiology were categorized and tabulated. Demographic details, clinical symptoms, and risk factors were also tabulated. IBM SPSS version 29 (IBM Corp., Armonk, NY) was used for the statistical analysis. Pearson’s correlation coefficient test was employed to assess the significance of discrepancies and to confirm or rule out a significant correlation.

## Results

Clinical findings

All the patients were adults, with ages ranging from 21 to 90 years, and the peak incidence occurred in the fifth decade. The male-to-female ratio was 2:1, and the results are presented in Table 1. 

**Table 1:** Age and sex distribution of patients with mucormycosis (n=74)

Age range	Number of patients	Males	Females
21-30	08	06	02
31-40	13	10	03
41-50	21	12	09
51-60	12	08	04
61-70	09	08	01
71-80	09	05	04
81-90	02	02	00
	n=74	51	23

The presenting symptoms included fever, headache, facial pain, and retro-orbital pain, with nasal congestion being the most common, followed by fever. A significant number of patients were diabetic, and most had received steroid therapy for COVID-19. The details are provided in Table 2.

**Table 2:** Presenting symptoms and risk factors

Symptom	A number of patients suffered
Fever	71
Headache	49
Nasal congestion	74
Facial and retro-orbital pain, diplopia	16
Black/blood-stained nasal discharge	45
Corticosteroid use (during COVID Infection)	71
Pre-existing diabetes	49

Histopathology findings

**Figure 1:**
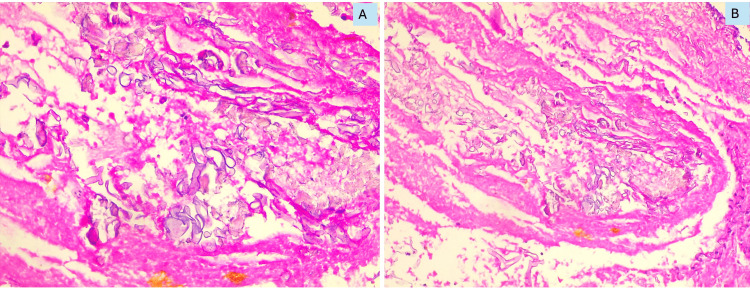
Photomicrograph of histopathology of Mucor stained with H&E A) Broad, aseptate, ribbon-shaped hyphal forms from a paranasal lesion (400X); B) Hyphal forms showing angioinvasion (200X). H&E: hematoxylin and eosin

All 74 suspected cases of mucormycosis were confirmed by histopathology, including six cases of mixed Mucor and Aspergillus infection. The histopathology of these lesions showed extensive necrosis, acute and chronic inflammatory infiltrates, and broad, aseptate, branching, ribbon-like fungal elements (Figure 1A). A few cases demonstrated angioinvasion (Figure 1B).

**Figure 2:**
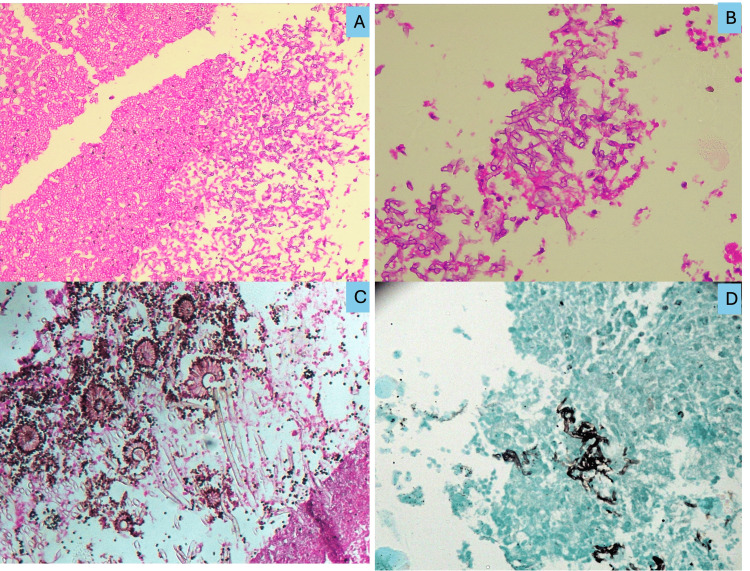
Photomicrograph of histopathology of Aspergillus A) Colony of Aspergillus in a mixed infection with Mucor and Aspergillus, H&E stain (200X). B) Narrow, branching hyphae of Aspergillus, H&E stain (400X). C) Numerous fruiting bodies of Aspergillus, H&E stain (400X). D) GMS stain demonstrating Mucor (400X). GMS: Gomori’s methenamine silver; H&E: hematoxylin and eosin

The six mixed Mucor and Aspergillus infections displayed both types of fungal elements, with Aspergillus forming large colonies of narrow, septate, and branching fungal hyphae (Figure 2A, 2B) with fruiting body formation (Figure 2C). Special stains such as Periodic acid-Schiff and GMS were used wherever necessary (Figure 2D).

Microbiology correlation

**Figure 3:**
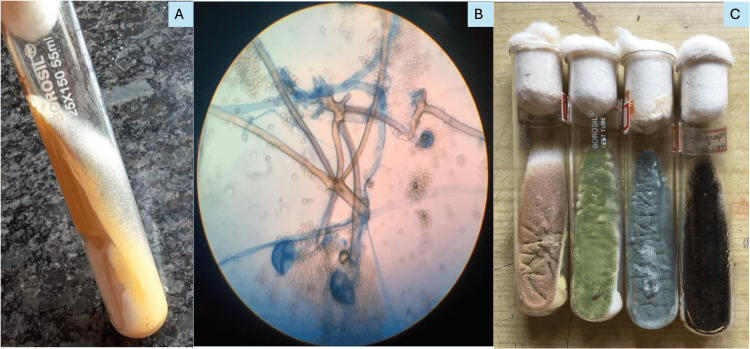
Photomicrograph from culture A) White and fluffy colonies of Mucor on SDA medium; B) Rhizopus species on LPCB mount; C) Aspergillus species on SDA medium. SDA: Saboraud's Dextrose Agar

Out of 74 Mucormycosis cases diagnosed on histopathology, 64 (86.5%) were confirmed by microbiology culture (Figure 3A). Among these 64 cases, Rhizopus was the most common fungal isolate (Figure 3B) seen in 43 (67.2%) cases, while Mucor was isolated in 21 (32.8%) cases. Lichtheimia* *species were not isolated in our study. A total of five (6.7%) cases of mixed Mucor and Aspergillus on histology turned out to be either Mucor (three) or Aspergillus (two) on culture and were included in the correlated category. Of the remaining 10 (13.5%) non-correlated cases, culture results yielded eight (10.8%) cases of Aspergillus(Figure 3C) and two (2.7%) cases of Candida. 

**Figure 4:**
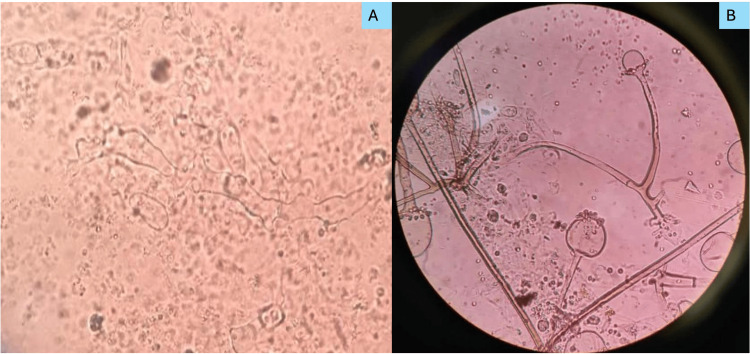
Photomicrograph of Mucor from KOH mount A, B) Two different cases showing broad and aseptate fungal hyphae.

All the KOH findings (Figures 4A, 4B) were consistent with respective culture isolates, and no discrepancies were noted. 

Discrepancies

Ten cases (13.5%) showed a morphological discrepancy between histopathology and microbiology. Eight cases (10.8%) diagnosed as Mucor on histopathology were identified as Aspergillus on culture. A single case was initially diagnosed as mixed Mucor and Aspergillus infection, and another as Mucor showed the growth of Candida on culture( 1.35% each). In all cases wherein discordant results emerged (between histopathology and microbiology), the patients were initially clinically diagnosed with Rhino-Orbito-Cerebral mucormycosis, and it was seen that in all those cases samples (swabs) were taken from the maxillary sinus and sent to the Microbiology lab, and biopsies from the maxillary sinus along with all the excised/debrided tissues were sent to the Histopathology lab. Slides with diagnostic discrepancies were reviewed, and then the diagnosis was re-confirmed. Molecular techniques could not be used in cases of discrepancies between histopathology and microbiology due to the unavailability of such techniques for diagnosing fungal infections in the institute. The results are presented in Table 3. 

**Table 3:** Comparison between histopathology findings and culture

Positive pathology finding	Microbiology culture	Count
Mucor (broad, aseptate, hyaline hyphae)	Mucor (Rhizopus or Mucor species.)	59 (79.7%)
Mucor (broad, aseptate, hyaline hyphae)	Aspergillus species	8 (10.8%)
Mixed Mucor and Aspergillus (broad, aseptate, hyaline hyphae seen along with narrow, septate hyphae)	Mucor (Rhizopus or Mucor species)	3 (4.05%)
Mixed Mucor and Aspergillus (broad, aseptate, hyaline hyphae seen along with narrow, septate hyphae)	Aspergillus species	2 (2.7%)
Mixed Mucor and Aspergillus (broad, aseptate, hyaline hyphae seen along with narrow, septate hyphae)	Candida	1 (1.35%)
Mucor	Candida	1 (1.35%)
		n=74

To assess the significance of discrepancies and to confirm or rule out a significant correlation, Pearson’s correlation coefficient test was used. It did not reveal a significant correlation in the case of histopathology and microbiological culture/KOH mount, but it did show a statistically significant correlation between microbiological culture and KOH mount (as all findings of KOH mount correlated perfectly with culture and LPCB mount findings), which was significant at the 0.01 level (r value=0.009). A correlation significant at p</=0.005/0.001 for all three diagnostic techniques (histopathological examination, KOH mount, and culture) would commonly indicate that the sample is either positive or negative for Mucor. However, it was not the case with histopathology and culture, as the correlation coefficient did not indicate any significant correlation (owing to the discrepancies noticed), and the results are depicted in Table 4.

**Table 4:** Correlation between culture and histopathology findings

		Histopathology findings
Microbiological culture findings	Pearson correlation	0.186
	Sig.(2-tailed)	0.112
	N	74

## Discussion

Mucormycosis is an uncommon opportunistic fungal infection that affects patients with predisposing conditions such as diabetes mellitus, steroid use, hematological malignancies, neutropenia, and organ transplantation [4]. During the second wave of COVID-19, India saw a significant surge in fungal infections, particularly rhino-orbito-cerebral fungal infections, with mucormycosis being the most common, followed by Aspergillosis [4]. This increase is multifactorial, including uncontrolled and unrecognized cases of diabetes, immune dysfunction, and hyperglycemia induced by COVID-19, and extensive use of corticosteroids and broad-spectrum antibiotics as a part of the treatment [5]. 

Mucor enters the body through inhalation/ingestion/inoculation in the form of spores, infecting primarily the nose and paranasal sinuses and tends to cause an invasive and potentially fatal infection [4]. The diagnosis of mucormycosis on histopathology is by the identification of broad, aseptate or pauci-septate, hyaline, and ribbon-like hyphae with right-angled branching. Angioinvasion with thrombosis, infarction, and necrosis are hallmarks of this infection [4,6,7]. This fungal infection is usually associated with significant inflammation, which may either be neutrophilic or chronic granulomatous in nature. Compared to non-COVID infections, COVID-19 produced more severe necrosis, angioinvasion, and higher fungal loads [7]. In contrast, the hyphal forms of Aspergillus* *are narrow (ranging from three to 12 microns in size), septate with dichotomous branching, and exhibit angioinvasion to a lesser extent when compared to Mucor. Candida exhibits both pathogenic yeast and pseudo-hyphal forms in tissues. However, the differentiation between various fungal species on histopathology is not always accurate [7]. The diagnosis of fungal infection involves a combination of KOH mount, histopathology, and culture, with culture being considered the gold standard for species identification. However, no single method is entirely accurate, each has its own merits and demerits [8].

The KOH mount is a useful, rapid, and cost-effective method for preliminary diagnosis but is limited by a lack of both specificity and sensitivity [8,9]. A fungal culture is regarded as the gold standard for specific species identification, but the disadvantages include false positives due to contamination or the presence of commensals, high cost, long duration, and, in some cases, lack of growth or sampling after the initiation of antifungal therapy, which results in false negatives [7-11]. Histopathology, on the other hand, is a relatively rapid investigation that allows for the direct visualization of fungi resulting in accurate species identification while providing crucial prognostic information such as extent and type of tissue damage in the form of angioinvasion, perineural invasion, necrosis, hemorrhage, and type of inflammation. However, morphological overlap between species hinders the diagnostic accuracy in biopsy specimens. The presence of fewer fungal elements, degenerative changes, necrosis, and inflammation can make the diagnosis difficult. Hence it is suggested that the histopathology report of a fungal infection should include a morphological description with a possible indication of the species, differential diagnosis, quantity of fungus, and the tissue reaction [8-14]. Histopathological features that positively impact culture positivity include a higher fungal load, tissue invasion, and necrosis, while angioinvasion appears to have a negative influence [15]. Table 5 provides a comparative analysis between KOH mount, culture, and histopathology. 

**Table 5:** Comparative analysis between KOH mount, culture, and histopathology

S.No	Feature	KOH	Culture	Histopathology
1	Principle	Direct examination of fungus using KOH	Isolation of fungus and growing in culture medium	Microscopic identification of fungus in tissue sections
2	Duration	Rapid	Long duration takes 2-3 weeks	24-28 hours
3	Cost	Cheap	Costly	Relatively cheap
4	Specificity	Low	High	High
5	Sensitivity	Low	Moderate	Moderate
6	Tissue reaction	No information	No information	Provide information about the type of inflammation
7	Prognostic information	Nil	Nil	Angioinvasion tissue necrosis fungal load

The present study showed a 100% (74/74) correlation of KOH mount with microbiology culture. The high correlation between KOH mount and culture isolates (100%) in our study is attributable to the processing of the same sample received by the microbiology department. On the other hand, the correlation between histopathology and culture in the present study is 86.4% (64/74), and the expected correlation between histopathology and culture is only 50% as suggested by previous studies [7]. These discrepancies between culture and histopathology could be due to different samples being sent to the two departments. Superficial swabs were sent to the microbiology laboratory, whereas much more invasive biopsy specimens were submitted to the pathology department for histopathological examination. Destruction of delicate fungal hyphae during culture processing such as grinding could be another reason for negative cultures [7,9]. 

Growth of Candida in culture in two cases diagnosed as Mucor (one) and mixed Mucor and Aspergillus (one) in the present study could be due to contamination during sampling or culturing. The presence of Mucor on histopathology and Aspergillus in culture, (8/74, 10.8%) indicates either contamination of the culture, co-infection, or misidentification of the species in biopsy [16]. In cases of fungal co-infection, one fungus tends to outgrow the other in culture (on SDA). This could also be a reason for discrepancies between histopathology and microbiology.

The limitations of the present study include its retrospective nature, the use of different tissue specimens from the same patient for microbiology and histopathology (although this is common practice in many settings, and we highlight the importance of using the same sample), a limited study period, and the lack of molecular techniques for diagnosing fungal infections at the institute.

## Conclusions

KOH mounts of samples are useful in diagnosing mucormycosis but are highly susceptible to human error, such as confusing background tissue debris with fungal elements, which can result in false-negative or false-positive results, reducing their practical accuracy. Fungal culture is a highly sensitive method that allows for the growth of pathogenic fungi, even with a low fungal load or the presence of very few viable spores or fungal elements in the sample. But it carries a risk of contamination and requires a long incubation time, occasionally giving negative findings (especially when there are no viable spores or if anti-fungal therapy has been initiated prior to sampling) making it unsuitable as the sole laboratory investigation for the timely diagnosis of mucormycosis. Histopathology supersedes culture in that it is faster and does not produce false positives. However, negative histopathology findings do not necessarily rule out a potential fungal infection, as negativity could be due to sampling error or difficulty in interpretation, even with special stains. Despite this, there is almost no chance of false positives (excluding visual errors) with histopathology, making it a highly specific investigation. Another advantage of histopathology is its ability to distinguish invasive and non-invasive fungal infections and pathogenic fungi from contaminants in biopsy specimens.

Our study emphasizes the ideal method to send the same specimens to both pathology and microbiology departments for accurate diagnosis and precise management. Inter-departmental collaboration is essential in centers where different samples are sent to different departments for better outcomes.
